# Influence of Intermediary Filling Material on Microleakage of Intracoronally Bleached and Restored Teeth

**Published:** 2009

**Authors:** Maryam Khoroushi, Atieh Feiz, Maysam Ebadi

**Affiliations:** * Associate Professor, Department of Operative Dentistry and Torabinejad Dental Research Center, School of Dentistry, Isfahan University of Medical Sciences, Isfahan, Iran; ** Assistant Professor, Department of Operative Dentistry and Torabinejad Dental Research Center, School of Dentistry, Isfahan University of Medical Sciences, Isfahan, Iran; *** Dentist, Isfahan University of Medical Sciences, Isfahan, Iran

**Keywords:** Adhesives, antioxidants, tooth bleaching, dressings, endodontically-treated teeth

## Abstract

**Background:**

Failure of composite restorations in terms of microleakage after intracoronal bleaching has been reported. The purpose of this study was to assess in vitro effect of sodium ascorbate and calcium hydroxide as intermediary filling materials to repair the microleakage associated with adhe-sive restoration following intracoronal bleaching.

**Methods:**

Sixty endodontically-treated incisors with access cavities extended to the cementoenamel junction in gingival margin were randomly divided into five equal groups. In group 1, cavities were restored by applying Single Bond and Z100 composite resin. In groups 2-5, 35% hydrogen peroxide gel was placed into the pulp chamber and sealed for 5 days. In group 2, teeth were then restored as in group 1. In groups 3 and 4, 10% sodium ascorbate gel and calcium hydroxide paste were applied in the pulp chamber for 40 hours, removed, rinsed and then, restored. In group 5, the cavities were incu-bated for 7 days and then, restored. Samples were thermocycled, immersed in basic fuschin, and sec-tioned. Dye penetration was scored using a stereomicroscope. Data were analyzed using Kruskal- Wallis and Mann-Whitney U tests (α = 0.05).

**Results:**

There was no significant difference in enamel margins (P = 0.163). In dentinal margins (P = 0.003), groups 1, 3 and 5 exhibited similar leakage patterns, each one of groups 1, 3 and 5 had sig-nificant differences with each one of groups 2 and 4.

**Conclusion:**

Intracoronal bleaching using 35% H2O2 gel increases the microleakage in dentinal margins. Application of the antioxidant agent or a seven-day delay following bleaching may improve the marginal integrity. Applying calcium hydroxide might jeopardize dentinal sealing.

## Introduction

After completion of intracoronal bleaching treatment to an extent that satisfies both the patients and their dentists, the access cavity should be restored using a permanent restoration material. The best choice in this case is composite-resin, which is bonded by means of acid-etch technique to enamel and dentin. Achieving a durable bond prevents exposure of the area to bacteria and other contaminants, guaranteeing tooth stability. Furthermore, strong restoration as well as safely sealed dentinal tubules are required for successful bleaching therapy.^1^,^2^ Some studies have shown that the bond strength of resin-based materials to bleached enamel and dentin is reduced provisionally.^3^,^7^ The peroxide residuals are believed to inhibit composite resin polymerization;^6^,^8^ it is less likely that changes in the enamel structure might influence resin composite adhesion.^9^,^10^ In any case, the appearance of a hybrid layer in bleached enamel is not as clear and regular as in enamel that has not been bleached.^11^ That is why access cavities in teeth that are bleached and restored by use of composite-resin sometimes show marginal leakage.^12^

Several methods have been proposed to neutralize the negative effects of hydrogen peroxide on the adhesion of resin composite bonded materials to the enamel and dentine of the access cavity in bleached teeth.^13^,^18^

Cvitko *et al* found that refreshing the margins through producing cavity bevel before acid-etching is effective in reducing the negative effects of hydrogen peroxide on adhesion.^13^ Rotstein *et al* used sodium hypochlorite to resolve the remnants of peroxide in the access cavity.^14^ Others have suggested pretreatment of enamel using dehydrating substances such as alcohol and acetone-containing adhesives to attain desirable results with regard to adhesion.^15^,^16^

Some authors reported that delaying bonding by at least 7 days can help maintain good adhesion of composites to dental tissues.^10^,^17^,^18^ Shinohara *et al* and Cavalli *et al* obtained the optimal bond of resin material to bleached dental hard tissues after a 3-week period.^19^,^20^ During this waiting period, the color of the bleached tooth should be stable. Recently, researchers declared that compromised bonding to bleached enamel can be reversed with sodium ascorbate, an antioxidant, either on dentin or enamel.^18^,^21^–^24^

One of the most important complications of bleaching is external root resorption (ERR), which in severe cases causes tooth loss. Kehoe found that bleaching agents reduced the pH in the cemento-enamel junction (CEJ), stimulating osteoclast activity, which in turn may initiate ERR.^25^ Temporary dressing of the pulp chamber with calcium hydroxide has been proposed to reverse pH and prevent ERR.^26^,^27^ Application of this material has also been recommended to function as a biological seal under the glass ionomer barrier, which serves only as a mechanical seal.^1^

A previous study claimed that the provisional application of calcium hydroxide suspension into the pulp chamber after completion of the bleaching procedure does not negatively impact the sealability of the composite materials used for final restoration of the access cavity.^28^

As marginal sealing is one of the key factors in the long-term success of endodontic, bleaching and restorative treatments, we sought to compare calcium hydroxide and sodium ascorbate as temporary intracoronal treatments for the microleakage of resin composite restorations in the bleached access cavity.

## Materials and Methods

In order to conduct this in vitro study, we selected 60 freshly extracted intact human maxillary incisors that had been extracted due to periodontal problems. Samples were stored in 0.2% thymol solution for two months before the study. Following a thorough cleaning of the teeth with a brush and pumice/water slurry, endodontic access was obtained using a round diamond bur in a high-speed hand-piece and cooled with air/water. Then, using fissure burs, access cavities were extended to CEJ areas; gingival margins were placed in the dentinal region. The root canals were cleaned, shaped and obturated with gutta-percha and root canal sealer (AH26, Dentsply, USA). The cervical third of the root canals were treated with the primer solution and sealed with a 2-mm layer of glass ionomer cement (Vitremer, 3M ESPE, Dental products, St. Paul, MN, USA) according to the manufacturer’s instructions (Table 1). The teeth were randomly divided into five groups of 12 specimens each and then, subjected to different treatments. In group 1 (negative control), all the access cavities were treated with the bonding agent (Single Bond, 3M ESPE, Dental products, St Paul, MN, USA) and were incrementally filled with composite resin (Z100, 3M ESPE, Dental products, St Paul, MN, USA) according to the manufacturer’s instructions (Table 1). Each increment was photocured for 40 seconds with a Coltoux 50 (Colten, Whaledent Inc, Mahwal, NJ, USA) light-curing unit.

**Table 1:** Restorative materials used in the study and mode of their applications according to the manufacturer instructions (3M ESPE).

Materials	Manufacturer’s instructions
Single Bond & Z100 composite resin	Etch for 15 seconds. Rinse with water spray for 10 seconds leaving tooth moist. Apply two consecutive coats of the adhesive with a fully saturated brush tip. Dry gently for 2 to 5 seconds. Light cure for 10 seconds. Apply Z100 composite resin. Light cure for 40 seconds.
Vitremer RMGI	Apply Vitremer primer for 30 seconds. Air-dry for 15 seconds. Light cure for 20 seconds. Mix the Vitremer powder and liquid in 2.5/1 ratio for 45 seconds. Apply the paste. Light cure for 40 seconds. Apply finishing gloss. Light cure for 20 seconds.

A 35% hydrogen peroxide gel (35% Hydrogen Peroxide, Opalescent endo, Ultra dent, USA) was placed into the pulp chamber. The cavities were sealed with cavit (Provis, Favodent karl Huber GmbH, Germany) for 5 days and then, incubated at 37°^C^ and 100% relative humidity. Then, the cavit was removed with round carbide burs. Cavities were washed with distilled water and prepared as follows:

In group 2 (Positive Control), after thorough rinsing, the cavities were all restored as in group 1. In group 3 (sodium ascorbate group), 10% sodium ascorbate gel was used for 40 hours. The cavities were sealed with cavit. The gel was then, washed thoroughly with distilled water and the cavities restored. In group 4 (calcium hydroxide group), calcium hydroxide powder (calcium hydroxide Pure, Merck, Germany) was mixed with saline solution in the ratio of one-scoop/one-drop. The paste was placed in the access cavities for 40 hours, the cavities were sealed with cavit and then, removed from the access cavities with a sharp spoon excavator. Cavities were washed with distilled water and restored. In group 5 (delayed bonding), a cotton pellet soaked in distilled water was placed into the pulp chamber and the cavity was sealed with cavit for 7 days. After this period, the cavit was removed using a carbide bur in a low-speed hand-piece and the cavity was washed with distilled water. The access cavities and pulp chamber were restored.

During each treatment and for 24 hours after restoration, all specimens were stored in the incubator at 37°C and 100% relative humidity. The restorations were polished by a composite polishing system (Enhance, 3M ESPE, Dental products, St Paul, MN, USA). Teeth were subjected to 500 thermal cycles, between 5°C and 55°C (Mp Based, KARA Co., Tehran, Iran).

The apices of all samples were then sealed; teeth surfaces were covered with two coats of nail varnish except for the restoration margins and 1 mm surrounding margins. Each group was immersed in a 2% basic fuschin solution for 24 hours. The nail varnish was removed and the teeth were longitudinally sectioned using a diamond disk. Microleakage in the incisal and gingival margins was examined by three cross-calibrated examiners under 32X magnification of a stereomicroscope (M6C-10, N9116734, Russia). The following standard scoring system was applied: 0, no dye penetration; 1, dye penetration that extended over up to half of the cavity wall depth; 2, dye penetration greater than half of the cavity wall depth. The obtained data were analyzed using non-parametric tests including the Kruskal-Wallis and Mann-Whitney U tests (α = 0.05).

## Results

Among the 5 groups under study, there was no statistically significant difference in microleakage of the enamel margin (P = 0.163) (Table 2). However, there were significant differences in the dentinal margins (P = 0.003) (Table 3). Pairwise comparisons of the dentinal groups based on the Mann-Whitney U test showed significant differences between groups 1, 3, 5 and groups 2, 4. Groups 1, 3 and 5 exhibited similar leakage patterns and each one of groups 1, 3 and 5 had significant differences with each one of groups 2 and 4.

**Table 2:** Microleakage distribution in enamel margins in the study groups.

Groups	0	1	2	Total
1	11 %91.7	1 %8.3	0 %0	12 %100
2	7 %58.3	4 %33.3	1 %8.3	12 %100
3	10 %83.3	2 %16.7	0 %0	12 %100
4	7 %58.3	4 %33.3	1 %8.3	12 %100
5	10 %83.3	2 %16.7	0 %0	12 %100
Total	45 %75	13 %21.67	2 %3.33	60 %100

P = 0.163

**Table 3:** Microleakage distribution in dentinal margins in the study groups.

Groups	0	1	2	Total
1	11 %91.7	1 %8.3	0 %0	12 %100
2	4 %33.3	8 %66.7	0 %0	12 %100
3	10 %83.3	2 %16.7	0 %0	12 %100
4	4 %33.3	7 %58.3	1 %8.3	12 %100
5	9 %75	3 %25	0 %0	12 %100
Total	38 %63.3	21 %35	1 %1.7	60 %100

P = 0.003

## Discussion

In this study, the gingival margins of the cavities were extended to the CEJ. Clinically, some root-filled discolored teeth that need intracoronal bleaching do not have intact enamel margins. This is due to the existence of caries, unintentional extension of the margin during preparation of the access cavity, trauma and wear, among other reasons.

Some studies suggested that the remaining oxygen following a bleaching procedure interferes with the resin bonding process.^29^ Restoration of a marginal seal may also be affected by bleaching remnants, increasing the microleakage of fluids and bacteria into the restored pulp chamber, ultimately leading to the failure of treatment.^29^–^31^

Complications of the walking bleach technique are attributed to the acidic pH of the bleaching reagent. Thirty percent H2O2 has a pH value of between 2 and 3. The low pH is undesirable because the whitening efficacy of unbuffered H2O2 is significantly lower than the effect of alkaline-buffered H2O2.^29^

In the current study, group 2 and 4 demonstrated increased microleakage in the enamel margin, but this increase was not significant. Provisional dressing with calcium hydroxide increased microleakage in the dentinal margins. In this regard, our results are not in accordance with a previous study.^28^ In the present study, all the cavities were washed with distilled water as a neutral irrigation medium. Demarco *et al* used an anionic detergent solution in their study to wash the cavities and reported no adverse effect on microleakage.^28^ There are probably precipitates of the material on the dentinal surface which are not completely removed even after etching with phosphoric acid.

Supporting the findings in this study, Mondelli *et al* suggested that although provisional dressings using calcium hydroxide, as well as their relationship to microleakage of resin composite restorations, remain to be elucidated, calcium hydroxide could undoubtedly remain in cavity margins and increase microleakage.^32^ Conversely, Piva *et al* reported that rinsing calcium hydroxide from the cavity using acid does not have a devastating effect on composite resin microleakage.^33^

In group 2, surface alterations and free oxygen may interfere with the formation of bonding and might be responsible for decreased adhesion, since this could prevent a close apposition between the adhesive agent and the tooth surface.^28^ Reduced microleakage in group 5 as compared to group 2 is related to the one-week delay in the placement of adhesive restorations. Others have reported^10^,^17^,^18^,^28^ that a delay in adhesive restoration could reverse the effect of the bleaching agents.

In contrast to findings reported by Demarco *et al,*^28^ increasing microleakage in dentinal margin in group 4 may imply that residual calcium hydroxide in the margins can not be removed completely, even using a total-etch bonding system. In fact, it seems that using calcium hydroxide in the dentinal part of the access cavity, which has a margin made entirely of enamel margin, would be expected to exert a buffering effect and reduce the likelihood of cervical resorption. However, it seems that simple rinsing after using calcium hydroxide in the bleached access cavity is not enough when the enamel margins are not intact and/or adequate, possibly leading to an increase in microleakage. Other methods, such as refreshing the margins or using calcium hydroxide as only a biological seal,^1^ are recommended in order to control the microleakage of restoration margins. However, further studies are necessary to conclusively determine the effect of calcium hydroxide on the adhesion of various materials to dental tissues.

## Conclusion

Using calcium hydroxide in the access cavity during the waiting period after intracoronal bleaching leads to increased dentinal microleakage. Before restoring the bleached access cavity, using sodium ascorbate or delaying bonding may improve marginal sealing.
